# Three-dimensional reconstruction by a computer-assisted surgery system for pediatric hydronephrosis caused by accessory renal artery compression: a single-center retrospective observational comparative case series

**DOI:** 10.3389/fped.2026.1809887

**Published:** 2026-07-21

**Authors:** Rongkun Zhu, Xiaoxuan Ma, Di Wang, Qian Dong, Jian Guo, Xiwei Hao

**Affiliations:** 1 Department of Pediatric Surgery, The Affiliated Hospital of Qingdao University, Qing Dao, China; 2 Cardiovascular Surgery Intensive Care Unit, The Affiliated Hospital of Qingdao University, Qing Dao, China

**Keywords:** accessory renal artery, children, computer-assisted surgery system, hydronephrosis, ureteropelvic junction obstruction

## Abstract

**Background:**

Ureteropelvic junction obstruction (UPJO) is the most common cause of pediatric hydronephrosis. For surgeons, a precise preoperative evaluation is essential, especially for hydronephrosis caused by accessory renal artery (ARA) compression. Conventional imaging modalities (ultrasound, magnetic resonance urography, computed tomography urography) often fail to identify the exact cause preoperatively, making precise surgical planning difficult. It is against this background that this case series is conducted. This is a single-center retrospective observational comparative case series to evaluate the clinical value of computer-assisted surgery system (CAS) three-dimensional (3D) reconstruction in children with hydronephrosis caused by ARA compression.

**Objective:**

To analyze and discuss the value of CAS 3D reconstruction in preoperative evaluation, surgical planning, and perioperative outcomes for pediatric hydronephrosis caused by ARA compression.

**Methods:**

This is a single-center retrospective observational comparative case series. A total of 263 children who underwent surgery for UPJO-related hydronephrosis were enrolled. By a retrospective review of preoperative imaging, medical records, and surgical documents, 20 patients with intraoperatively confirmed ARA compression were identified and divided into a 3D reconstruction group (*n* = 13) and a control group (*n* = 7). Preoperative etiological diagnosis, operative time, intraoperative blood loss, length of hospital stay, and complications were compared between the two groups.

**Results:**

All patients met surgical indications. Conventional imaging did not detect ARA compression preoperatively, whereas CAS 3D reconstruction identified ARA compression in all 13 patients of the 3D group. Operative time and intraoperative blood loss showed significant between-group differences [108 (98–128) vs. 128 (124–132) min, *P* = 0.002; 5 (4–8) mL vs. 8 (5–15) mL, *P* = 0.007]. Length of hospital stay was comparable [9 (7–11) vs. 9 (7-12) days, *P* = 0.838].

**Conclusion:**

In this single-center retrospective observational comparative case series, CAS 3D reconstruction visualized ARA compression preoperatively and provided comprehensive anatomical information for surgical planning, while conventional imaging failed to detect such compression. It shows a significant association with favorable perioperative metrics in pediatric patients with ARA-related hydronephrosis.

## 1 Introduction

Hydronephrosis in children has various causes, with ureteropelvic junction obstruction (UPJO) being the most common. UPJO can be classified as intrinsic, extrinsic, or secondary. The prevalence of UPJO caused by crossing renal vessels varies substantially by pediatric subgroup. Rooks and Lebowitz (2001) found such vascular obstruction in 11% of prenatally detected UPJO but in 50% of symptomatic surgically treated children (1, 2).

In children, the accessory renal artery (ARA) is often slender and difficult to detect on ultrasound, CT, or magnetic resonance urography (MRU). Failure to identify an ARA preoperatively may increase surgical risk and operative time. Therefore, accurate preoperative mapping of the ARA origin, course, crossing point relative to the ureter, and the renal segment supplied is clinically important.

Computer-assisted three-dimensional (CAS 3D) surgical reconstruction systems—such as 3DR Labs, 3D-IRCAD, Synapse 3D, and Hisense CAS—have been widely used in hepatobiliary, oral, and thoracic surgeries. Hisense CAS has been widely used in pediatric surgical planning and enables precise segmentation of organs and three-dimensional visualization of vascular structures. This study retrospectively evaluates the impact of preoperative 3D reconstruction using Hisense CAS on surgical outcomes in children with ARA-compression hydronephrosis.

## 2 Materials and methods

### 2.1 Study design

This was a single-center retrospective observational comparative case series conducted at the Department of Pediatric Surgery, The Affiliated Hospital of Qingdao University, Qingdao, China. The study analyzed only existing routine clinical and imaging data without additional interventions, examinations, or radiation exposure to patients.

### 2.2 Ethics approval, registration, and consent

Ethical approval was granted by the Institutional Review Board of The Affiliated Hospital of Qingdao University (approval number: QYFYWZLL30161). Prospective trial registration was not applicable because this was a retrospective review of anonymized clinical data and not a prospective interventional trial. The ethics committee waived the requirement for individual informed consent because of the retrospective, non-interventional, and anonymized nature of the study. Written informed consent for publication was obtained from the legal guardians of all patients.

### 2.3 Inclusion and exclusion criteria

#### 2.3.1 Inclusion criteria

1. Children aged 0–18 years who underwent surgical repair for UPJO-related hydronephrosis between January 2015 and December 2024.
2. Availability of complete preoperative imaging, operative notes, and clinical follow-up data.
3. Intraoperatively confirmed ARA compression as the cause of obstruction.

#### 2.3.2 Exclusion criteria

1. Incomplete clinical or imaging records.
2. Major associated congenital anomalies interfering with surgical outcome assessment.
3. Emergency surgery or intraoperative conversion unrelated to ARA anatomy.

### 2.4 Participant flow

Initial cohort: 263 children with UPJO hydronephrosis.

Excluded: 243 patients without intraoperatively confirmed ARA compression or incomplete data.

Final sample: 20 patients with ARA-related hydronephrosis.

Group allocation: 3D reconstruction group (*n* = 13): Preoperative CAS 3D reconstruction performed.

Control group (*n* = 7): No preoperative 3D reconstruction.

### 2.5 Group allocation rationale

Group assignment was non-random and based on clinical complexity, surgeon preference, and institutional workflow evolution. Preoperative 3D reconstruction was gradually adopted for anatomically complex or suspected vascular cases, while earlier reported cases or those with simpler anatomy were managed without 3D planning.

### 2.6 Baseline characteristics

Data on baseline parameters such as age, sex, laterality, hydronephrosis severity, surgical approach, ARA position, associated anomalies, and attending surgeon were collected and compared between the two groups (Table 1). No significant baseline imbalances were identified.

**Table 1:** Statistics of basic information of enrolled patients.

Variable	Total (*n* = 20)	3D reconstruction group (*n* = 13)	Control group (*n* = 7)	*p*-Value
Age, median (range), years	8.5 (2.0–15.0)	9.0 (2.5–15.0)	7.0 (2.0–12.0)	0.317
Sex, *n* (%)				1.000
Male	14 (70.0)	9 (69.2)	5 (71.4)	
Female	6 (30.0)	4 (30.8)	2 (28.6)	
Laterality, *n* (%)				1.000
Left	12 (60.0)	8 (61.5)	4 (57.1)	
Right	8 (40.0)	5 (38.5)	3 (42.9)	
Hydronephrosis severity, *n* (%)				0.642
Moderate	9 (45.0)	5 (38.5)	4 (57.1)	
Severe	11 (55.0)	8 (61.5)	3 (42.9)	
ARA position relative to the ureter, *n* (%)				0.613
Anterior	14 (70.0)	10 (76.9)	4 (57.1)	
Posterior	6 (30.0)	3 (23.1)	3 (42.9)	
Attending surgeon, *n* (%)				1.000
Surgeon A	11 (55.0)	7 (53.8)	4 (57.1)	
Surgeon B	9 (45.0)	6 (46.2)	3 (42.9)	

For continuous variables, the median (range) is adopted; the number of cases used for categorical variables (%); categorical variables: two-sided Fisher’s exact test, continuous variables (age): Mann–Whitney *U* test.

### 2.7 Technical details for 3D reconstruction

1. CT protocol: Routine contrast-enhanced CT; slice thickness ranged from 0.625 to 2.00 mm, with a spacing of 5 mm (acquired in cortical, nephrogenic, and excretory phases).
2. CAS platform: Hisense CAS 3.0 (Qingdao Hisense Medical Technology Co., Ltd., Qingdao, China).
3. Workflow: DICOM data imported into the CAS system; automated segmentation of kidney, ureter, and renal vessels; manual correction by two experienced pediatric surgeons; final quality check by a senior attending surgeon.
4. Radiation exposure: No additional imaging or radiation exposure was required; reconstruction used only standard preoperative CT data.
5. Intraoperative validation: 3D anatomical findings were directly compared with intraoperative visual inspection by the operating surgeon.

### 2.8 Endpoint definitions

Primary endpoint: Operative time (min).

Secondary endpoints: Intraoperative blood loss (mL), postoperative hospital stay (days), perioperative complications.

### 2.9 Statistical analysis

Because of the small sample size, continuous variables were presented as median (range). All statistical analyses were performed using IBM SPSS Statistics for Windows, version 26.0 (IBM Corp., Armonk, NY, USA).

All *p*-values presented in Tables 1, 2 were recalculated based on the original individual patient raw data and using the following methods: Continuous variables: Mann–Whitney *U* test; Categorical variables: Fisher’s exact test; a *p*-value <0.05 was considered statistically significant. No other statistical method was used for this comparison.

**Table 2:** Perioperative outcomes between the two groups.

Outcome	3D reconstruction group (*n* = 13)	Control group (*n* = 7)	*p*-Value
Primary endpoint			
Operative time, median (range), and minutes	108 (98–128)	128 (124–132)	0.002
Secondary endpoints			
Intraoperative blood loss, median (range), and mL	5 (4-8)	8 (5-15)	0.007
Postoperative hospital stay, median (range), and days	9 (7–11)	9 (7–12)	0.838
Perioperative complications, *n* (%)	0	0	-

Data are expressed as median (range) or as number of cases (%); A *p*-value <0.05 was considered statistically significant. Intraoperative blood loss was estimated from original anesthesia and operative records (3D reconstruction group 5.2 ± 1.3 mL; control group 7.8 ± 3.2 mL). The Mann–Whitney *U* test was used for between-group comparison.

## 3 Results

A total of 20 children with ARA-related hydronephrosis were included in the study: 13 in the 3D reconstruction group and seven in the control group. Baseline characteristics were balanced between the two groups (Table 1). The median age of the cohort was 8.5 (2.0–15.0) years; 14 were males and six were females. All patients had moderate or severe hydronephrosis. Most ARAs arose from the aorta and crossed anteriorly or posteriorly to the ureter.

Associated congenital anomalies included mild renal dysplasia (*n* = 2), ureteral polyp (*n* = 1), and nephrolithiasis (*n* = 2). No horseshoe kidney, duplex collecting system, ectopic kidney, contralateral renal anomaly, or vesicoureteral reflux was present. The distribution of associated anomalies was similar between the groups.

### 3.1 Hisense CAS 3D reconstruction findings

**Figure 1:**
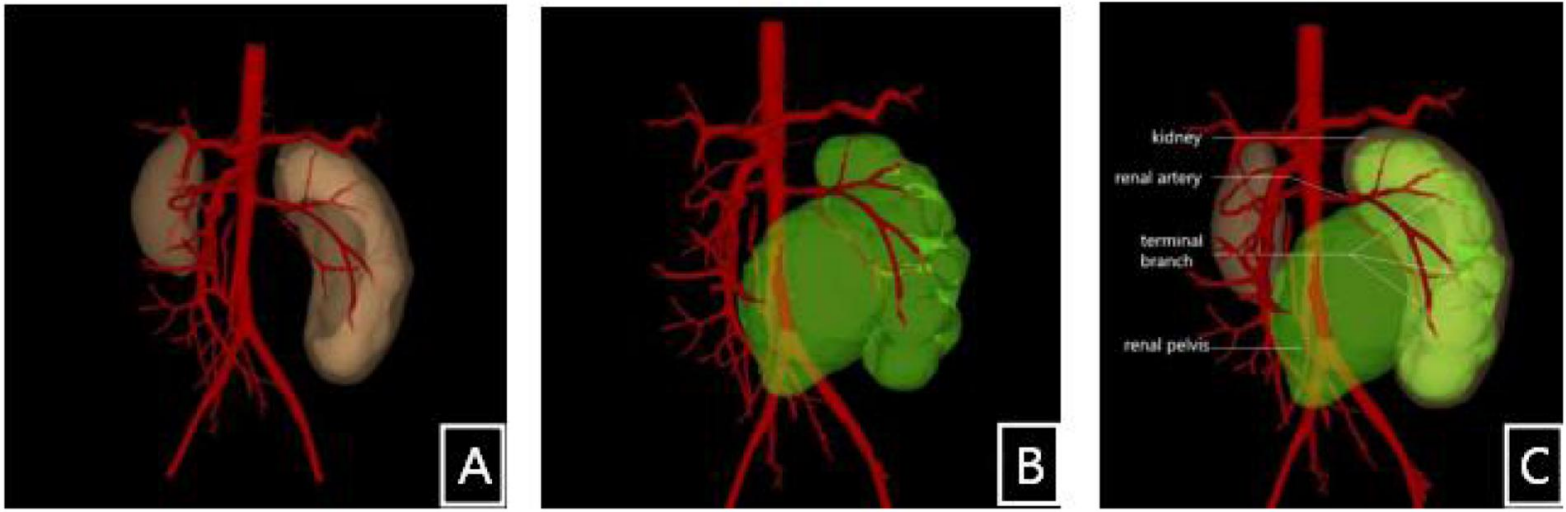
CAS 3D reconstruction of UPJO. **(A)** Individual 3D images of the vessels and kidneys are arranged to clearly display the vascularization and innervation of the terminal branches within the kidney. **(B)** A 3D image illustrates the extent of pyelonephrosis and more clearly defines the site of ureteropelvic obstruction. **(C)** 3D stereoscopic images of the vessels, ureters, kidneys, and renal pelvis, reassembled to represent UPJO.

**Figure 2:**
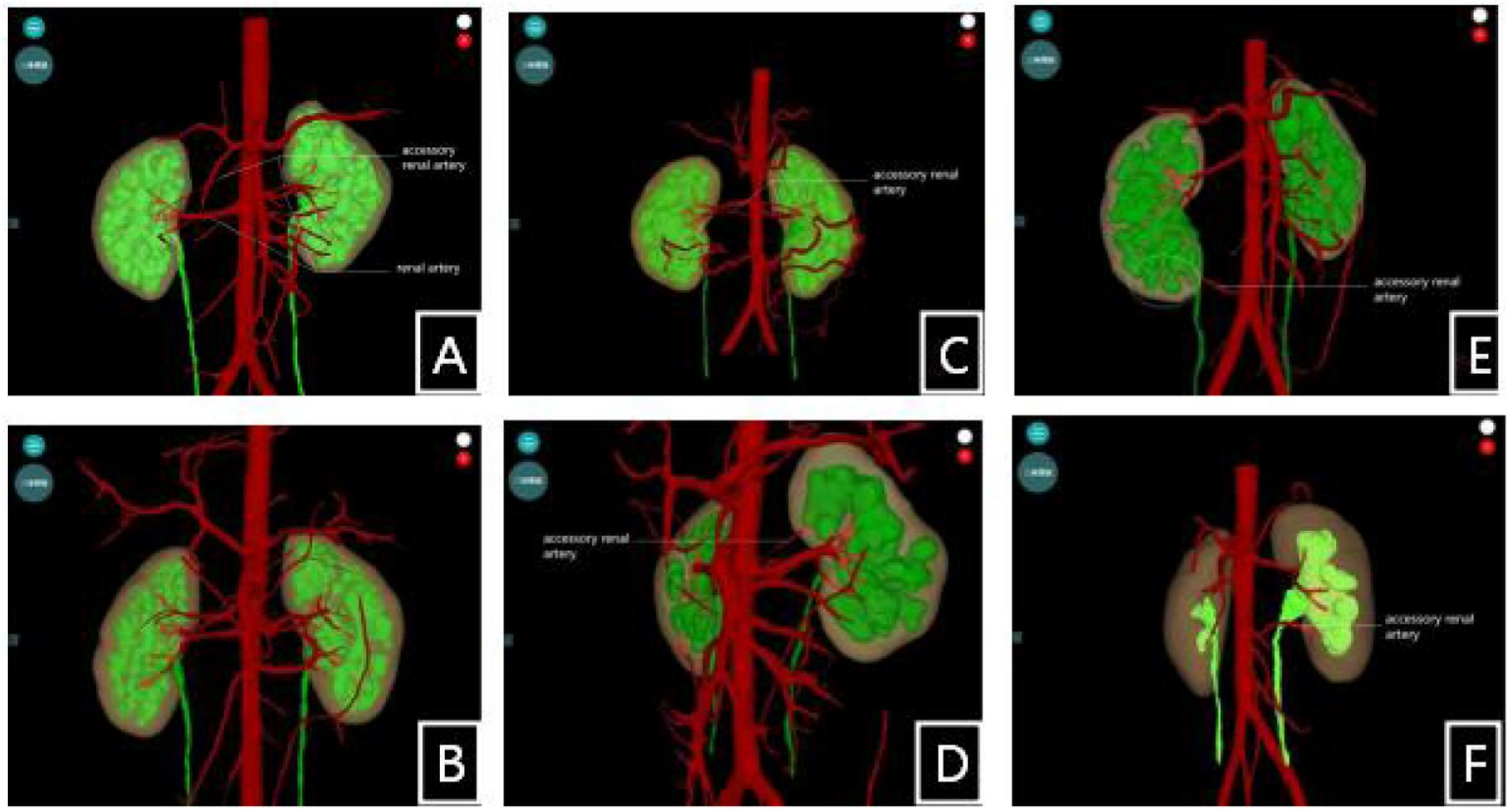
CAS 3D-reconstructed images showing the origin and course of the ARA. **(A–F)** The sequential panels demonstrate the anatomical alignment and branching pattern of the accessory renal artery.

**Figure 3:**
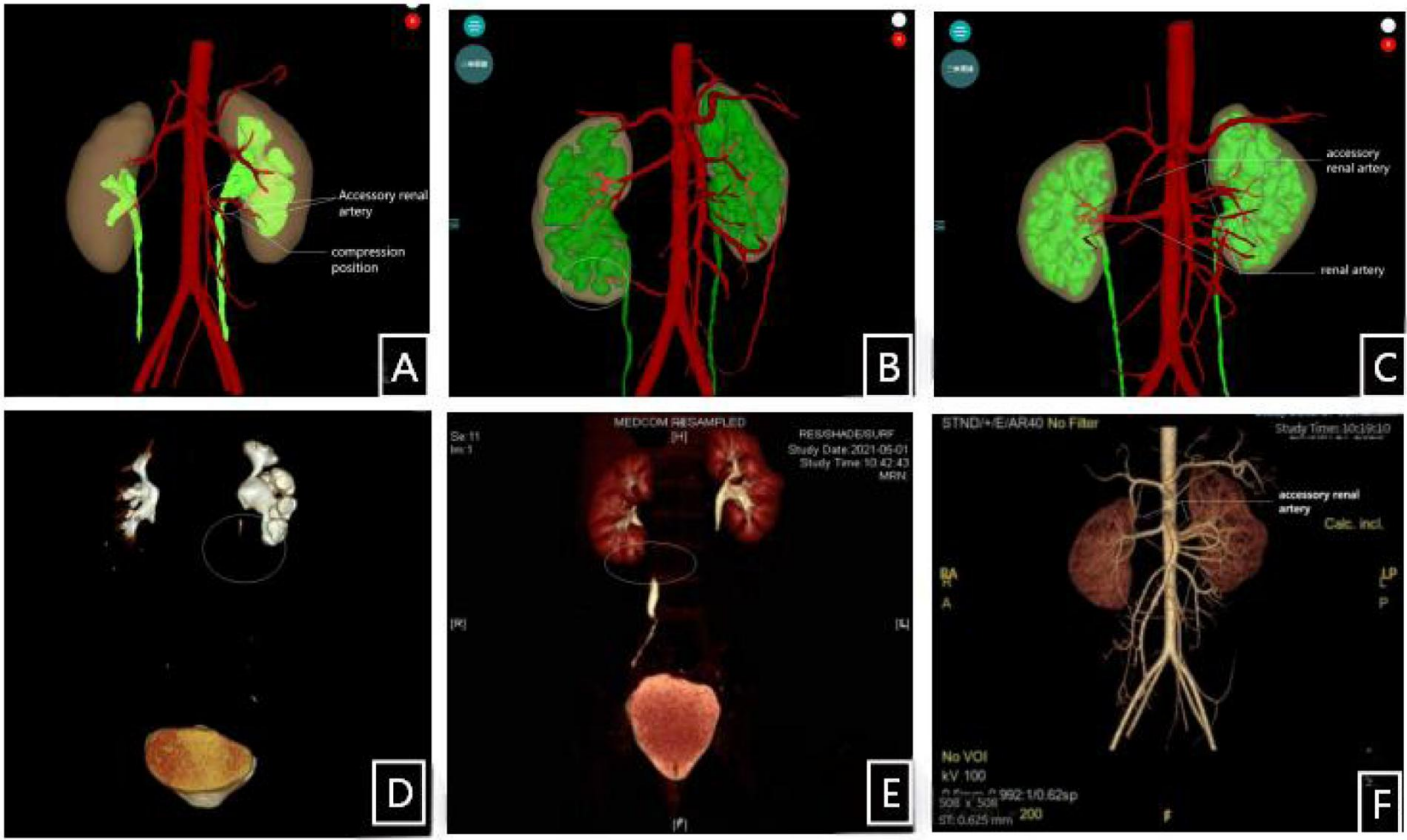
A comparison of CAS 3D reconstruction, CTU, and CTA images. **(A–C)** CAS 3D images clearly visualize the thin pediatric ARA. **(D–F)** Conventional CTU and CTA fail to clearly delineate the ARA.

ARA compression as the etiology of obstruction was confirmed intraoperatively in all 20 patients. The 3D reconstruction model clearly delineated the origin, course, ureteral compression site, and renal segment supplied by the ARA in each case (Figures 1–3). Virtual surgical rehearsal and quantitative evaluation were performed preoperatively using the 3D model. For anterior crossing ARAs, virtual vascular transposition and division were both simulated during preoperative planning to support decision-making regarding vessel preservation, although only vessel-preserving techniques (transposition/vascular hitch) were ultimately performed in all cases. This objective evaluation provided a scientific basis for treatment planning, while the actual surgical management in all 3D reconstruction group patients consisted of vessel-preserving procedures (vascular hitch or transposition) without ARA division.

### 3.2 Comparative analysis of CAS 3D reconstruction versus computed tomography urography and CT Angiopraphy (CTA)

**Figure 4:**
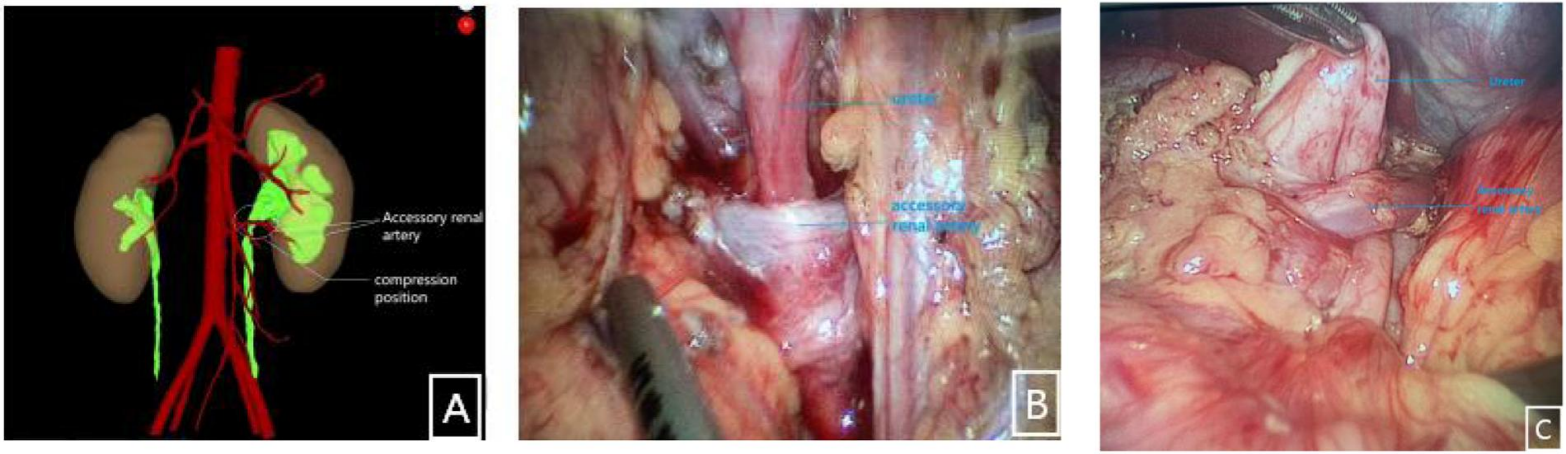
Concordance between CAS 3D reconstruction and intraoperative findings. **(A)** A preoperative 3D-reconstructed image. **(B and C)** An intraoperative anatomical view showing consistent alignment with the 3D model.

Hisense CAS 3D images provided an integrated, interactive, and stereoscopic display that incorporated information from computed tomography urography (CTU) and CTA. Compared with CTU and CTA, 3D reconstruction clearly visualized the thin pediatric ARA that was poorly visualized or undetected by conventional imaging (Figure 3). Anatomical details on 3D reconstruction were highly consistent with intraoperative findings (Figure 4).

### 3.3 Histopathological findings

**Figure 5:**
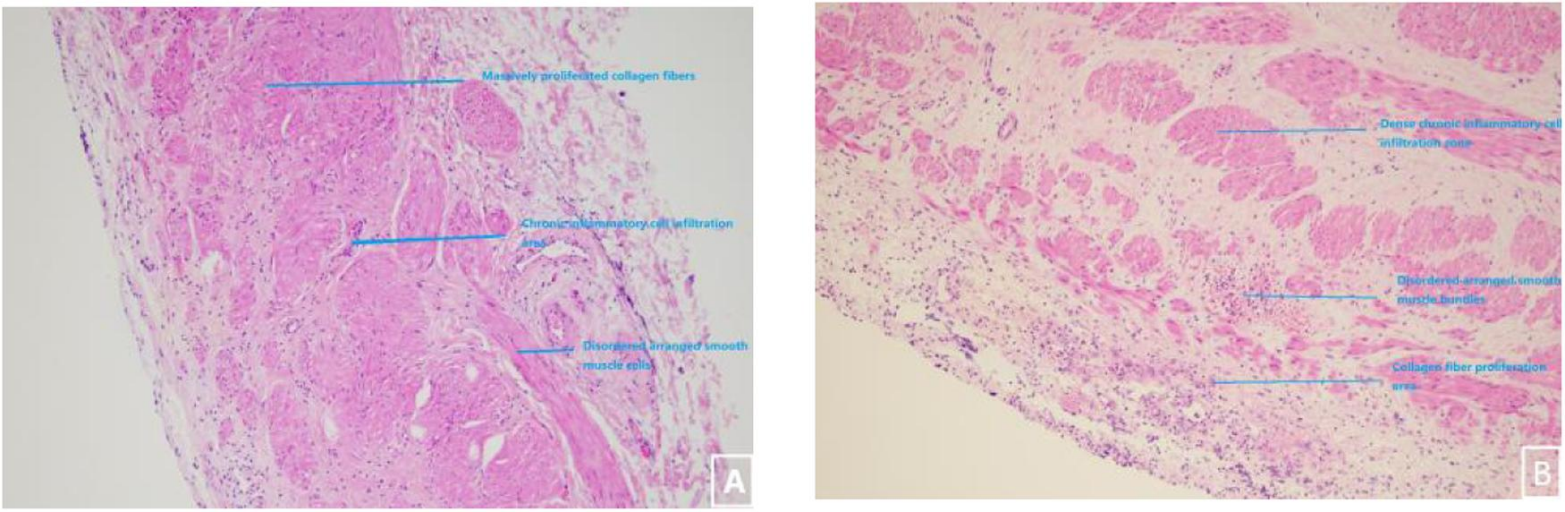
A histopathology of resected UPJ stenotic segments. **(A)** ARA-compressed UPJ: smooth muscle disarray, fiber fragmentation, and diffuse collagen deposition. **(B)** Non-ARA-compressed UPJ (intrinsic stenosis): chronic inflammatory infiltration, disorganized muscle bundles, and focal collagen hyperplasia (HE staining,  × 10).

A histological examination of the resected ureteropelvic junction (UPJ) stenotic segment from patients with ARA-induced obstruction showed disorganization of the smooth muscle layer, fragmentation of muscle fibers, and extensive interstitial collagen deposition. In contrast, the resected UPJ stenotic segment from patients with non-ARA-induced obstruction (e.g., primary intrinsic UPJ stenosis) showed dense chronic inflammatory cell infiltration, disorganized smooth muscle bundles, and focal collagen fiber hyperplasia (Figure 5).

### 3.4 Perioperative outcome comparison

Perioperative outcomes are presented in Table 2.

1. Operative time was significantly different between the two groups (*P* = 0.002, Mann–Whitney *U* test).
2. Intraoperative blood loss was significantly different between the groups (*P* = 0.007, Mann–Whitney *U* test).
3. Postoperative hospital stay was comparable between the groups (*P* = 0.838).

### 3.5 No perioperative complications occurred in either group

CAS 3D reconstruction clearly visualized the origin, course, ureteral crossing point, and supplied renal segments of the ARA, with excellent concordance with intraoperative findings. Furthermore, in the present cohort, all patients underwent vessel-preserving management; no ARA division was performed.

## 4 Discussion

This single-center retrospective observational comparative case series evaluated the clinical value of preoperative 3D reconstruction using a CAS 3D in children with hydronephrosis secondary to ARA compression. The key finding was that preoperative CAS 3D reconstruction was associated with shorter operative time and less intraoperative blood loss in this small cohort, without increasing hospital stay or complication rate. The difference in intraoperative blood loss reached statistical significance, but the absolute median difference was only 3 mL. Its clinical significance remains uncertain given the similar perioperative outcomes in both groups. But these results support that CAS 3D reconstruction can improve anatomical understanding and facilitate precise surgical planning for pediatric ARA-related hydronephrosis.

UPJO is the most common cause of pediatric hydronephrosis. As a relatively rare extrinsic etiology, ARA compression is difficult to identify preoperatively using conventional imaging modalities such as ultrasound, CTU, and MRU. In clinical practice, unrecognized ARA may increase the risk of intraoperative vascular injury (3), prolong exploration time, or lead to incomplete resolution of obstruction, resulting in postoperative recurrence (4). In the present study, conventional imaging failed to detect ARA compression preoperatively, while CAS 3D reconstruction clearly showed the origin, course, crossing site with the ureter, and supplied renal segments of ARAs, which was highly consistent with intraoperative findings.

Compared with CTU and CTA, CAS 3D reconstruction provides an integrated and interactive stereoscopic model that allows rotation, transparency adjustment, and anatomical measurement (5). Preoperative CAS 3D reconstruction may help surgeons evaluate the origin, course, crossing point, diameter, and supplied renal segment of the ARA, thereby supporting individualized surgical planning (6, 7). In the present cohort, all patients underwent vessel-preserving management, and no ARA division was performed. When a crossing artery supplies an appreciable renal territory, vessel-preserving management, including dismembered pyeloplasty with vessel transposition, cephalad translocation, or vascular hitch, should be considered (8, 9). Although selected forms of vessel sacrifice have been reported, including rare crossing-vessel division (10) and crossing-vein division with preservation and cephalad relocation or upward transposition of the crossing artery (11, 12), routine division of an accessory renal artery is not supported by the available evidence. Therefore, any decision to divide a vessel should be individualized and based on intraoperative findings, vessel type, supplied renal territory, renal perfusion, and functional significance. If vessel division is performed, postoperative evaluation for renal ischemia, segmental perfusion loss, renal function, and recurrent obstruction should be reported. Currently, the concept of precision surgery is pursued globally, and the preoperative delineation of the origin of the ARA, its alignment, its crossing position with the ureter, and the renal segment supplied by its terminal end has become the focal point in the field of pediatric urology (13, 14), as well as an important factor influencing the success of laparoscopic (15) pyeloplasty for UPJO (16).

Several limitations of this study should be acknowledged. First, this was a retrospective, non-randomized comparative case series with a small sample size (13 vs. 7), which may lead to potential selection bias. Second, group assignment was based on clinical complexity and surgeon preference rather than random allocation. Third, the single-center design may limit the generalizability of the conclusions. Fourth, because 3D reconstruction was gradually adopted during the study period, chronological bias and surgeon learning-curve effects may also have influenced operative time and blood loss. Therefore, the present results are associative rather than causal.

In conclusion, in this small retrospective cohort, preoperative CAS 3D (17) reconstruction was associated with a significantly shorter operative time and less intraoperative blood loss in children with hydronephrosis caused by ARA compression. These results support that CAS 3D reconstruction may enhance anatomical understanding and may facilitate precise surgical planning for pediatric ARA-related hydronephrosis. Although a statistically significant difference in intraoperative blood loss was observed (median difference = 3 mL), the absolute gap was minimal, and the clinical relevance of this finding remains uncertain. Further studies are needed to verify the long-term efficacy of the procedure. Further large-sample, multicenter, or matched cohort studies are warranted to validate these findings.

## Data Availability

The datasets presented in this study can be found in online repositories. The names of the repository/repositories and accession number(s) can be found in the article/Supplementary Material.
